# Review of D-Shape Left Ventricle Seen on Magnetic Resonance Imaging (MRI) or Computed Tomography (CT), Similar to the Movahed Sign Seen on Cardiac Gated Single-Photon Emission Computed Tomography (SPECT) as an Indicator for Right Ventricular Overload

**DOI:** 10.3390/jcm14176041

**Published:** 2025-08-26

**Authors:** Daniel McCoy, Mohammad Reza Movahed

**Affiliations:** 1 College of Medicine, University of Arizona, Phoenix, AZ 85004, USA; 2 Sarver Heart Center, University of Arizona, Tucson, AZ 85724, USA

**Keywords:** myocardial perfusion imaging, gated SPECT, D-shape LV, D-shape left ventricle, right ventricular overload, pulmonary embolism’ pulmonary hypertension, cardiac magnetic resonance imaging, cardiac MRI, computed tomography, cardiac CT

## Abstract

The “Movahed sign” refers to the presence of a D-shaped left ventricle on cross-sectional cardiac imaging due to interventricular septal flattening seen during cardiac gated single-photon emission computed tomography (SPECT) This phenomenon arises from significant right ventricular (RV) pressure or volume overload, which pushes the septum toward the left ventricle (LV) and distorts the LV’s normally circular profile into a “D” shape. However, the prevalence and incidence of similar findings during cardiac Magnetic Resonance Imaging (MRI) or computed tomography (CT) are not known. The goal of this study was to perform a literature search focusing on the “Movahed sign” or D-shaped left ventricle in the context of cardiac MRI and CT. Databases searched included PubMed and Google Scholar, and reference lists of relevant articles were reviewed. The echocardiography literature was also consulted for foundational concepts of septal flattening. Key data on pathophysiology, imaging features, clinical correlations, and prognostic significance were extracted.

## 1. Introduction

**Figure 1:**
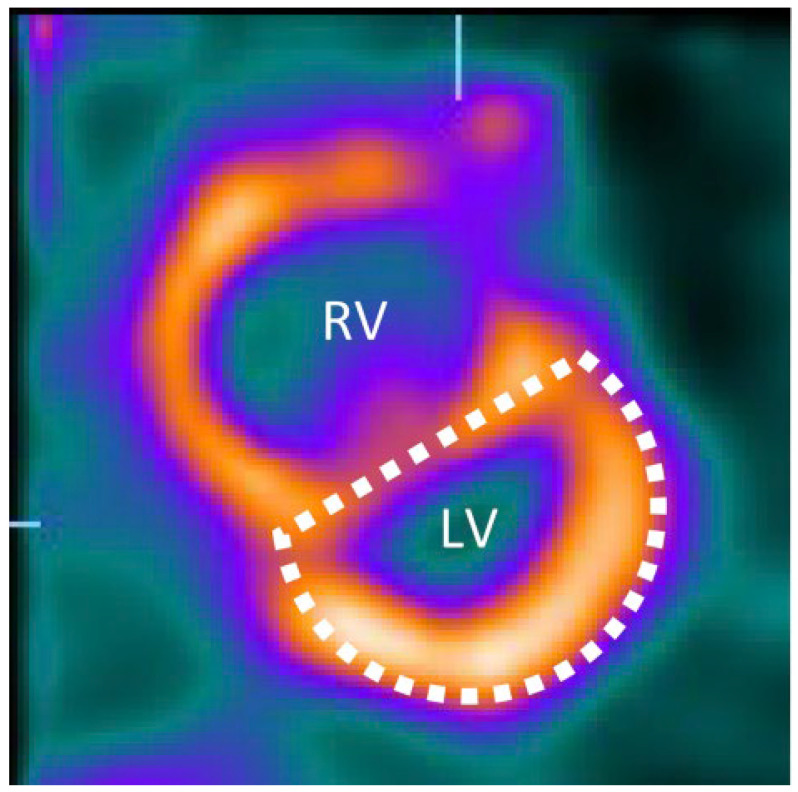
Movahed’s sign on short-axis myocardial perfusion SPECT images at rest. Reproduced from Ref. [8] under CC BY license.

**Figure 2:**
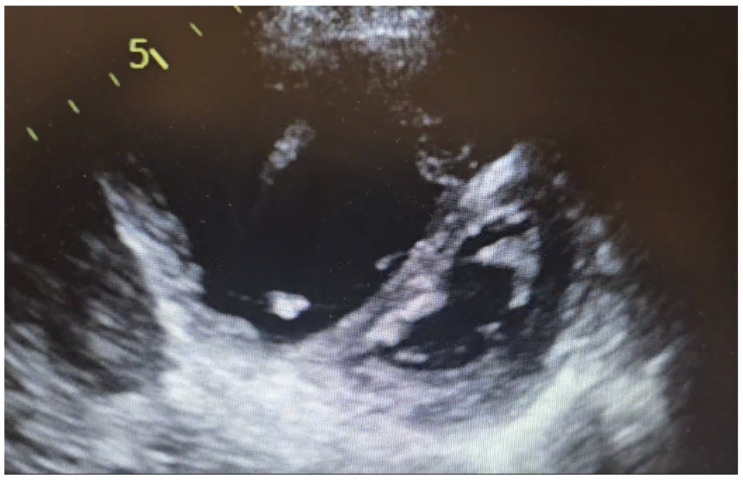
Flattening of the interventricular septum in a 79-year-old patient with severe PH (original).

The “Movahed sign” refers to the presence of a D-shaped left ventricle on cross-sectional cardiac imaging due to interventricular septal flattening [1] Figure 1. This phenomenon arises from significant right ventricular (RV) pressure or volume overload, which pushes the septum toward the left ventricle (LV) and distorts the LV’s normally circular profile into a “D” shape [1,2,3]. While septal flattening on echocardiography has long been recognized as a hallmark of RV overload, (Figure 2) Movahed et al. first described this sign on gated myocardial perfusion SPECT (single-photon emission computed tomography) images in 2005 [2]. In honor of that work, the D-shaped LV on nuclear imaging is often termed Movahed’s sign. The same pathophysiologic sign can be appreciated on cardiac Magnetic Resonance Imaging (MRI) and computed tomography (CT), modalities which provide higher anatomical detail. However, the prevalence and incidence of similar findings during MRI or CT are not known. This review provides a comprehensive overview of the Movahed sign, with emphasis on its pathophysiology, imaging characteristics on MRI and CT, diagnostic utility and differential diagnosis, epidemiology, associated conditions, clinical implications, and controversies. High-quality studies, including original research, case reports, and reviews, are highlighted to inform cardiologists and cardiac imaging specialists of the current understanding and clinical relevance of this imaging sign.

## 2. Methods

A literature search was performed focusing on the “Movahed sign” or D-shaped left ventricle in the context of cardiac MRI and CT. Databases searched included PubMed and Google Scholar, and reference lists of relevant articles were reviewed. Priority was given to peer-reviewed studies, including the original descriptions by Movahed and colleagues, subsequent validation studies, case reports, and review articles in cardiology and radiology. The echocardiography literature was also consulted for foundational concepts of septal flattening. The search encompassed publications from the mid-2000s (when the sign was first described in SPECT) to the present. Key data on pathophysiology, imaging features, clinical correlations, and prognostic significance were extracted. The findings are synthesized below. This study is a narrative review. While a structured literature search was performed, this review does not adhere to formal systematic review protocols such as PRISMA or PICO frameworks. Only human studies were included, except where veterinary models were briefly cited to illustrate specific imaging concepts. Because “Movahed sign” is a term rooted in nuclear cardiology, some older sources describe it in SPECT; however, this review emphasizes the sign’s manifestations on MRI and CT and extrapolates nuclear/echo findings where applicable.

## 3. Pathophysiological Basis and Origins

Interventricular septal position is governed by the balance of pressures and volumes between the RV and LV. In normal physiology, the septum bows mildly toward the RV (which is lower pressure) and maintains a convex shape toward the RV throughout the cardiac cycle [3,4]. Significant RV overload, due to either high pressure (e.g., pulmonary hypertension) or high volume (e.g., large left-to-right shunt), disturbs this balance. The septum is pushed leftward, flattening against the LV cavity and sometimes even bowing into it [3,5]. The result is a D-shaped LV on short-axis imaging. Mechanistically, acute or chronic RV pressure overload, such as acute massive pulmonary embolism or chronic pulmonary arterial hypertension, tends to cause septal flattening throughout systole (and often diastole), whereas pure RV volume overload (e.g., an atrial septal defect without severe pulmonary hypertension) causes flattening more prominently in diastole [3,5]. The phenomenon is one aspect of ventricular interdependence; because both ventricles share the septum and pericardial space, an enlarged or over-pressurized RV will encroach on LV filling geometry [6,7].

Originally, septal flattening in RV overload was noted on echocardiography and recognized as an indicator of pulmonary hypertension (PH) or cor pulmonale. Movahed and colleagues were the first to systematically report this sign on gated SPECT myocardial perfusion scans [2]. In their 2005 observational study, eight of eight patients with septal flattening on SPECT had confirmed RV pressure overload (pulmonary artery systolic pressures 42–52 mmHg on echo) from causes like COPD, atrial septal defect (ASD), pulmonary embolism (PE), or sleep apnea [2]. They noted that SPECT could surprisingly visualize this D-shaped LV, even when echocardiography did not. In their series, only 50% of cases showed the flattening on echo, whereas it was evident on all SPECT images [2]. This finding, coupled with increased RV tracer uptake and RV dilation on SPECT, was highly specific for RV overload [2]. Subsequent reports reinforced that a D-shaped LV on gated imaging correlates strongly with elevated RV afterload; Murarka and Movahed’s 2010 review urged nuclear cardiologists to routinely assess septal shape on perfusion scans as an “obvious” clue to conditions like PH or acute PE [1]. Because Movahed’s work brought attention to the sign in the nuclear imaging community, the eponym “Movahed’s sign” became associated with the D-shaped LV on gated scans (Figure 1).

Importantly, the same biophysical sign is observable on MRI and CT, which directly depict septal morphology. These modalities are not constrained by the limited RV visualization of SPECT. Thus, while the name “Movahed sign” originated in nuclear cardiology, the imaging manifestation, a flattened interventricular septum, has broad relevance across cardiac imaging. In MRI and CT, it is typically just referred to as a septal flattening or D-shaped ventricle, but we will use the term Movahed sign here when discussing analogous findings.

## 4. Imaging Characteristics on MRI

**Figure 3:**
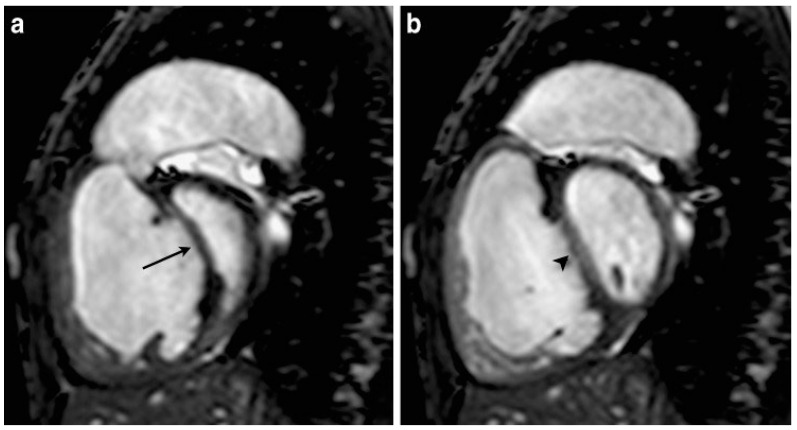
An 18-year-old man with severe idiopathic PH. Short-axis steady-state free precession cine MR image at early diastole (**a**) and end diastole (**b**). Arrow showing septal shift toward left ventricle causing a D-shape left ventricle. Reproduced from Ref. [23] under CC BY license.

Appearance on Cardiac MRI: On cardiac MRI, the Movahed sign is best appreciated in axial short-axis cine images (typically the mid-ventricular slice at the level of papillary muscles, Figure 3). In a normal patient, the LV short-axis cross-section is roughly circular. In the setting of significant RV overload, the interventricular septum flattens and the LV assumes a D shape, with the septum forming the straight segment of the “D” [1,9,10]. This configuration may be present in systole, diastole, or both, depending on the nature of the overload. For example, in chronic pressure overload from PH, one often sees the septum flattened or even bowing into the LV throughout systole and diastole (persistent D shape), whereas in pure volume overload (e.g., an isolated ASD with normal pressures), the septum might appear normal in systole but flatten in diastole when the RV is most distended. MRI cine sequences allow for analysis of this temporal behavior. In practice, an experienced observer will note a D-shaped LV immediately on visual assessment of the cine loops. Quantitatively, MRI researchers use the eccentricity index (EI), the ratio of the LV diameter parallel to the septum vs. perpendicular to the septum, to gauge the degree of septal flattening [11,12]. An EI > 1 (i.e., septum flattened) indicates a D-shaped LV, and higher values correspond to more severe flattening. An EI ≥1.2 on MRI has been linked to adverse outcomes in PH, underlining the clinical significance (more on that in Prognostic Implications) [13].

MRI can also depict secondary signs accompanying the Movahed sign. The RV is often enlarged and may have hypertrophied walls if the overload is chronic. In systole, the septum in RV pressure overload not only flattens but can exhibit paradoxical motion (moving towards the LV in systole rather than towards the RV). This can be seen on cine MRI and indicates RV dysfunction. MRI phase-contrast or 4D flow sequences might visualize abnormal interventricular flow patterns if a shunt is present or confirm the absence of leftward septal bowing in normal states [14,15,16,17,18,19]. Importantly, MRI also allows simultaneous assessment of the RV size, function, and pulmonary artery dimensions, which provide context for the D-shaped LV. In PH, one often sees an enlarged main pulmonary artery and RV alongside the septal flattening. In acute cases like massive PE, MRI (if performed) might show a D-shaped LV plus tricuspid regurgitation (TR) or septal hypokinesis. MRI can also assess TR severity using 2D phase-contrast and 4D flow MRI sequences, which may support the diagnosis of RV volume overload.

MRI Examples: Short-axis steady-state free precession (SSFP) cine images from patients with PH classically demonstrate the D-shaped LV. For instance, in an MRI study of patients with RV pressure overload, the septal curvature was found to inversely correlate with measured pulmonary artery pressures: the more flattened the septum (i.e., larger D-shape index), the higher the pulmonary pressure [20,21]. In a representative case (from a veterinary model of an ostium primum ASD with secondary PH), MRI in end diastole clearly showed a flattened septum and D-shaped LV, indicating elevated RV pressure [22].

On MRI, one must distinguish true septal flattening from artifact. Misregistration or off-axis images can sometimes give the illusion of a straight septum. Thus, the proper image plane (true short-axis) is important. Real septal flattening usually comes with other corroborating MRI findings (RV enlargement, high RV mass, etc.). Overall, the Movahed sign on MRI is a readily recognized marker of RV strain. Its presence should prompt the imager to carefully evaluate for causes of PH or acute RV pressure changes if not already known.

## 5. Imaging Characteristics on CT

**Figure 4:**
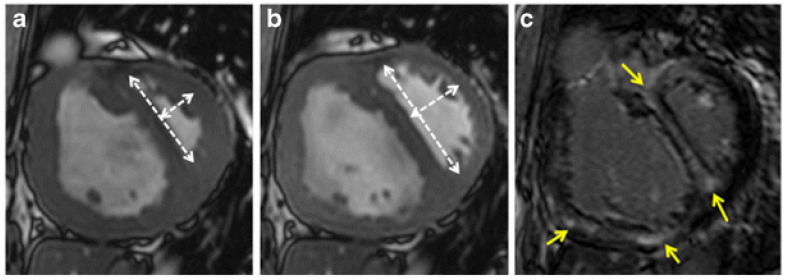
Short-axis CT images of end-diastolic cine (**a**), end-diastolic cine (**b**) and late gadolinium enhancement (LGE) (**c**) in a 49-year-old patient with atrial septal defect and Eisenmenger syndrome. (**a**) Systolic EI is 2.1. This result indicates the presence of PAH (mean PAP > 25 mmHg). (**b**) Diastolic EI is 2.2. This result indicates the RV systolic dysfunction (RVEF < 40%). (**c**) Yellow arrows represent LGE, which indicates myocardial fibrosis in RV and septum. Reprinted/adapted with permission from Ref. [11]. Copyright 2016, Springer.

Appearance on CT: On cardiac CT, the Movahed sign is most often encountered on contrast-enhanced chest CT scans done for PE or on dedicated cardiac CT scans when RV pressure is high (Figure 4). It can be seen in axial or four-chamber reformatted views. In a standard transverse (axial) CT slice through the ventricles (usually obtained in the context of a CT pulmonary angiogram), a normal interventricular septum is curved toward the RV. With significant RV pressure elevation, the septum flattens or bows toward the LV, and the cross-section of the LV becomes D-shaped [24]. Radiologists often simply describe this as “septal bowing” on CT. CT assessment of the septal configuration has high specificity for severe RV strain. Reviews have reported that interventricular septal bowing on CT had ~100% specificity for identifying RV dysfunction in acute pulmonary embolism, albeit with a sensitivity around ~30% [24,25]. In other words, if you see a D-shaped LV (septal flattening) on a PE-protocol CT, it is a very specific sign that the patient’s RV is under significant strain or pressure (often indicating a high-risk PE or massive clot burden). Conversely, its absence does not rule out RV strain. Key CT correlates of the Movahed sign include enlargement of the RV (commonly measured as an RV/LV diameter ratio >1 on CT) and dilation of the pulmonary artery. In fact, CT findings of RV/LV > 1 and septal flattening often go hand in hand, and both indicate right-sided pressure overload [26]. Some CT studies define septal flattening qualitatively, while others use the same EI measurement as echo/MRI (by measuring LV dimensions on axial slices). In either case, the presence of an abnormally straight or leftward-bowed septum is considered an ominous CT sign in acute PE.

CT Examples: In acute PE, a classic CT sign of severity is when the interventricular septum bows into the LV. For example, one study of PE patients found that an “abnormal septal morphology” on CT was significantly more frequent in high-risk (hypotensive) PE cases than in others [27]. Another analysis noted that leftward septal bowing on CT correlated with large clot burden and was associated with higher mortality [24,28,29]. Typically, radiologists evaluating a PE CT will comment on the septal contour: if flattened or deviated, they will raise concern for RV failure. In chronic PH, if a patient undergoes a non-gated chest CT for another reason, one may incidentally see a D-shaped LV as well, though this is more commonly evaluated via echocardiography or MRI in practice. On ECG-gated cardiac CT (such as CT angiography of coronary arteries in a patient who also has PH), one might capture the septal flattening during specific phases. Gated CT images in end systole for a PH patient would likely show the septum midline or bowing leftwards (D-shaped LV). Additionally, CT can show secondary signs like enlargement of the main pulmonary artery (>30–35 mm) and IVC contrast reflux, which often accompany a D-shaped LV in cases of acute cor pulmonale [30,31,32].

One point to note is that CT images are typically one snapshot in time (for non-gated scans) or a specific phase (in gated scans), so differentiating systolic vs. diastolic flattening as one might on echo/MRI is not usually possible on CT. However, multiphase cardiac CT can show the change in septal shape through the cycle if needed. Usually, though, the presence of septal flattening on any phase of CT is enough to indicate abnormal physiology.

In summary, on CT, the Movahed sign manifests as flattening or reversal of the septal curvature towards the LV. It is a striking and specific indicator of RV strain in acute settings like PE and can be recognized as part of the radiologic signs of right heart dysfunction (along with RV enlargement and PA dilation). It essentially mirrors what is seen on MRI and echo, just on a different modality.

## 6. Diagnostic Utility, Differential Diagnosis, and Clinical Contexts

The Movahed sign is a highly specific imaging marker of RV overload, particularly pressure overload [1]. When seen on MRI or CT, it should prompt evaluation for PH, acute PE, RV outflow tract obstruction (e.g., critical pulmonary stenosis), or significant volume load (e.g., large ASD or RV failure with expansion). In the appropriate clinical setting, its presence can clinch the diagnosis of acute cor pulmonale. On cardiac MRI, the sign confirms RV hemodynamic compromise and supports a diagnosis of PH, especially when other indicators (e.g., TR velocity or catheter data) are unavailable. Among patients with pulmonary arterial hypertension (PAH), the degree of septal flattening correlates with mean pulmonary artery pressure (mPAP) and pulmonary vascular resistance, aiding in noninvasive assessment of disease severity.

The Movahed sign is highly specific for true RV overload. In Movahed’s original study and subsequent analyses, it was rarely a false positive; nearly all patients with the sign had underlying RV pressure or volume pathology [1]. Its inclusion in SPECT interpretation improves detection of occult PH or RV dysfunction [33]. In CT for PE, septal flattening has near-100% specificity (though low sensitivity) for RV dysfunction [24,25], making it a valuable rule-in sign for significant RV strain.

The timing of septal flattening can help differentiate pressure versus volume overload. This is primarily described in echocardiography but is also applicable to cine MRI. Diastolic-only flattening suggests RV volume overload (e.g., ASD or severe TR), while flattening in systole or throughout the cardiac cycle indicates pressure overload (e.g., PE or PH) [1,2,3,4,5]. For example, diastolic flattening in an elderly patient may suggest an undiagnosed ASD or high-output state, whereas persistent systolic flattening points toward PH.

**Differential Diagnosis and Clinical Context:** When one encounters a flattened interventricular septum on imaging, the differential diagnosis primarily centers on causes of RV overload. The cardiac conditions most commonly associated with this sign include the following:

- **Pulmonary hypertension:** This is by far the most common cause in practice of a chronic D-shaped LV. This can be idiopathic PAH or PH secondary to left heart disease, lung disease, or chronic thromboembolism. In PH, septal flattening is usually present in both systole and diastole on imaging. Additional MRI clues might include RV hypertrophy and dilated pulmonary arteries. In advanced PH, MRI and CT often demonstrate a flattened septum [30,34]. Clinically, these patients present with dyspnea on exertion and signs of right heart failure. The sign correlates with elevated RV systolic pressure (>60 mmHg) and typical RV remodeling in PH [35].
  ○ **Left heart disease leading to PH:** Severe left heart failure or mitral valve disease can cause secondary PH. In such cases, one might see a D-shaped LV on imaging despite the primary issue being left-sided, because long-standing post-capillary PH causes RV pressure elevation [36,37,38]. For example, severe mitral stenosis can lead to a D-shaped LV due to resulting PH and RV overload (though the condition also affects LV filling). In advanced heart failure with preserved EF and resultant PH, septal flattening can also occur.
  ○ **Cor Pulmonale from Lung Disease:** Chronic lung diseases (COPD, interstitial lung disease) often cause gradual RV pressure overload (cor pulmonale). In advanced COPD with resting PH, CT scans can show a D-shaped LV along with large central pulmonary arteries [39,40,41,42]. MRI could similarly show it. These patients will have other signs like enlarged pulmonary artery diameter on CT > 30 mm, indicating PH [43].
  ○ **Chronic Thromboembolic Pulmonary Hypertension (CTEPH):** This treatable cause of PH can also show the D-shaped LV on imaging. In CTEPH, one might find the septal flattening on MRI plus visualized organized clots in pulmonary arteries on MR or CT [44,45].
- **Acute pulmonary embolism:** This is a leading cause of acute septal flattening. In massive PE with obstructive shock, the Movahed sign may be seen on a CT angiogram as septal bowing [25]. Echo would show McConnell’s sign (RV free-wall hypokinesis with apical sparing) alongside the D shape [46,47]. Clinically, these patients may be hypotensive, tachycardic, with acute right heart strain on ECG. The Movahed sign on CT in this context is an emergency finding, indicating likely need for aggressive therapy (thrombolysis or embolectomy).
- **Right ventricular infarction:** An acute RV myocardial infarction can cause acute RV failure and increased RV end-diastolic pressure, potentially leading to septal shift. The septum might move dyskinetically rather than purely flatten, and RV infarction rarely causes significant septal flattening unless there is concomitant PH [48,49].
- **Right Ventricular Cardiomyopathy or Failure:** End-stage RV failure from any cause can produce a D-shaped LV if pressures equalize abnormally [3,5,40]. In advanced arrhythmogenic right ventricular cardiomyopathy (ARVC), significant RV dilation and systolic dysfunction can produce septal flattening and a D-shaped LV, particularly when RV pressures are elevated. However, this remains a relatively uncommon etiology compared to PH or acute PE and is usually accompanied by other overt features of RV failure.
- **Volume overload of the RV,** such as large ASD (especially ostium secundum ASDs with significant left-to-right shunt) or severe chronic TR. These typically produce diastolic septal flattening. An ASD, for instance, will enlarge the RV, and in diastole, the septum flattens due to the increased RV filling volume [3,5,13]. In systole, if pulmonary pressures are normal, the LV may pop back to round. MRI with phase contrast can quantify a shunt if present (Qp:Qs ratio) [5,50].
- **Congenital Heart Disease:**
  ○ Atrial Septal Defect: Large, long-standing ASDs can lead to RV volume overload and, if Eisenmenger physiology develops, pressure overload [40,51]. An unrepaired ASD may show diastolic flattening; if pulmonary pressures rise (Eisenmenger syndrome), systolic flattening appears as well [3,5]. In pediatric patients with big ASDs, MRI is sometimes used to quantify shunts and will show the D-shaped LV at end diastole [5,50,52].
  ○ Ventricular Septal Defect (VSD) with pulmonary hypertension: A large VSD initially causes volume overload of both ventricles. If pulmonary vascular disease ensues, RV pressure rises, and a D shape can appear [3,53,54,55]. In infants with large VSDs, one might see this on echo if they develop early PH [5,12,53].
  ○ Tetralogy of Fallot: Before repair, these patients have RV pressure equal to systemic (due to the VSD and RV outflow obstruction), but since the pressures are balanced by the VSD shunt, the septum may not flatten much in diastole. After repair, if pulmonary regurgitation leads to volume overload, diastolic flattening can occur. However, this is a less typical scenario for a persistent D-shaped LV [55,56,57].
  ○ Ebstein’s anomaly: Severe cases have an enlarged atrium and functional ASD; if there is significant TR and an ASD, the RV overload (volume) could cause a D shape in diastole [55,58,59].
- **Constrictive pericarditis:** Interestingly, constrictive pericarditis can also cause a form of septal flattening, though it is usually a transient “septal bounce” rather than a persistent D shape. In early diastole, the septum may shift leftward with inspiration (ventricular interdependence) and produce a brief D-shaped LV. The context of constriction (pericardial thickening, respiratory variation on Doppler) differentiates it [19,60,61]. Thus, constriction is in the differential for any unusual septal motion. In MRI or CT, one clue favoring constriction is that septal flattening occurs only in early diastole and varies with respiration, as opposed to fixed flattening in all phases for true RV overload.
- **Acute Respiratory Distress Syndrome (ARDS) or Acute Asthma:** Severe acute lung processes can cause acute PH and RV strain (acute cor pulmonale). In ARDS, 20–25% develop acute cor pulmonale; CT may show a D-shaped LV, though it is not always obtained [62,63,64,65]. Echocardiography more often demonstrates septal flattening in severe ARDS [66,67].
- **Miscellaneous:** Large right-sided tumors or masses (like a huge RV fibroma or aneurysm) theoretically could compress the LV and mimic septal flattening, but this is exceedingly rare. Likewise, septal hypertrophy in hypertrophic cardiomyopathy may distort LV shape without producing true septal flattening.

In practice, when one sees the Movahed sign on MRI/CT, the differential is usually narrowed by the clinical scenario (e.g., known PH vs. acute dyspnea with possible PE vs. known ASD). Ancillary imaging findings help: a PE on CT is diagnosed by the clots in pulmonary arteries. Chronic PH on MRI might show delayed gadolinium enhancement in the RV insertion points, etc.

**Pitfalls:** One must be careful not to overcall septal flattening. Mild septal deviation can be subjective; for a definitive Movahed sign, the septum is visibly flattened. Minor interventricular septal flattening could potentially appear if the LV is underfilled (e.g., hypovolemia) while the RV is normal, but a true D shape is usually not seen just from underfilling. Technical factors like off-axis imaging or motion artifact on CT can simulate a straight septum. In left bundle branch block or ventricular pacing, the asynchronous contraction can cause a septal “flash” that momentarily alters septal position, but this is a dynamic movement, not a sustained D shape [68,69].

The Movahed sign’s primary diagnostic value lies in its specificity for RV overload, whether due to pressure or volume. Its recognition on MRI or CT should prompt focused evaluation of the right heart, including assessment for PH, PE, or intracardiac shunts. The pattern of septal flattening can help differentiate pressure versus volume overload and guide further diagnostic testing if the underlying etiology is not already established.

Importantly, the sign typically signifies an advanced stage of RV involvement. It is rarely seen in mild disease and usually appears when RV systolic pressure approaches systemic levels, as in severe PH or submassive to massive PE. Clinically, the presence or progression of a D-shaped left ventricle over time can indicate worsening RV strain. In patients without a prior diagnosis of PH, it should trigger evaluation for chronic thromboembolic disease or other causes of RV overload. In post-operative or ICU settings, a newly observed Movahed sign may signal acute RV failure, prompting timely interventions such as ventilator adjustment or pulmonary vasodilator therapy.

## 7. Prevalence and Epidemiological Insights

The prevalence of the Movahed sign depends on the population and imaging modality. It is not a common finding in general populations but is frequently seen among patients with significant PH or acute PE:

- **In Pulmonary Hypertension:** Septal flattening is a common feature in moderate to severe PH. Echocardiographic studies have documented D-shaped LV in a substantial fraction of PH patients; Bossone et al. reported that systolic flattening of the interventricular septum was observed in 90% of patients with primary PH, with the majority having pulmonary artery systolic pressures greater than 60 mm Hg [70]. On MRI, almost all patients with severe PH will exhibit some degree of septal flattening. Several studies support this finding. For instance, Pandya et al. demonstrated a strong inverse correlation between septal curvature and mPAP in pediatric patients with PH, with correlation coefficients of −0.81 and −0.85 at baseline and during vasodilator testing, respectively [20]. Similarly, Sciancalepore et al. found that septal curvature values progressively decreased with increasing severity of PH and correlated well with invasive pressures, with r-values ranging from 0.78 to 0.79 [71]. Roeleveld et al. also reported a significant relationship between septal curvature and systolic pulmonary arterial pressure, with a correlation coefficient of 0.77 [21]. Essentially, by the time pulmonary pressure approaches systemic levels, the D-shaped LV emerges [3,72,73]. Thus, epidemiologically, among patients with World Health Organization Group 1 PAH, the Movahed sign could be present in over half of those in NYHA class III–IV or with severe hemodynamics. However, exact prevalence varies by cutoff used and patient mix.
- **In Acute Pulmonary Embolism:** The prevalence of septal bowing on CT in acute PE corresponds to the proportion of patients with massive or submassive PE (those causing RV strain). Kim et al. reported that septal bowing was observed in 32.7% of patients with massive or submassive PE and in 5.5% of patients with small PE [27]. Similarly, Araoz et al. found that septal bowing was associated with an increased risk of short-term death, with an odds ratio (OR) of 1.98 in univariate analysis and 1.97 in multivariate analysis, although the sensitivity was low and interobserver variability was fair [28]. Beenen et al. also highlighted the prognostic value of septal bowing in their study, emphasizing its association with adverse outcomes [74].
- **General Population/Incidental:** In the general population, a D-shaped LV is essentially never seen unless a person has one of the pathologies above. Incidental detection on a non-cardiac CT scan (e.g., a trauma CT where the heart is visible) would be rare and should prompt evaluation for unrecognized PH or an acute event. Epidemiologically, one might say the sign has a prevalence approaching 0 in healthy individuals and 0 in patients with normal RV pressure. In patients with any form of significant RV overload, the prevalence rises with the severity of overload. For instance, among patients with COPD and cor pulmonale, echo studies often show septal flattening in about 20–30% of moderate COPD and up to 50% in very advanced COPD with resting PH [39].

In epidemiologic terms, the sign is strongly associated with conditions of higher mortality. In PH registries, those with septal flattening tend to be the sickest subset. In acute PE cohorts, those with septal bowing on CT have higher short-term mortality (in one study, 7.7% 30-day mortality in patients with RV/LV > 1 and septal bowing vs. <1% in those without these signs) [75]. So, while not prevalent in the overall heart disease population, when present, it usually portends a significant underlying pathology. A summary of representative imaging findings across clinical cases is provided in Table 1.

These cases highlight the diversity of underlying etiologies, from PH and TR to congenital shunts and pericardial disease, that can result in septal flattening. They also emphasize the importance of interpreting septal morphology in the context of clinical history, phase of cardiac cycle, and additional imaging findings such as RV dilation or pulmonary artery enlargement.

## 8. Clinical and Prognostic Implications

The Movahed sign has significant prognostic and clinical implications due to what it represents, a strained RV. Its presence usually indicates that the RV is under substantial stress and may be decompensating or at high risk of failure. Key implications include the following:

- **Indicator of Disease Severity:** In chronic conditions like PH, a D-shaped LV is a marker of severe disease [3,78]. Patients with septal flattening generally have more advanced NYHA functional class and worse hemodynamics than those without. For instance, septal flattening on baseline echo/MRI has been associated with lower exercise capacity and higher likelihood of clinical worsening [79]. One study of severe TR patients found that those with a D-shaped LV (EI ≥ 1.2) had significantly worse survival than those without flattening [13]. Thus, it portends a poor prognosis if not corrected. In PH trials, a reduction in septal flattening (improvement in EI toward normal) is sometimes seen with effective therapy, correlating with improved output. Persistent or worsening D shape despite therapy might suggest the need for escalated treatment or transplant evaluation.
- **Risk Stratification in Acute PE:** The identification of septal bowing on a CT or echo in acute PE places the patient in a higher risk category. Guidelines for PE management consider signs of RV dysfunction as criteria for “submassive” PE (which may warrant thrombolytic therapy if there is evidence of myocardial injury). Septal bowing is significantly more common in patients with high-risk PE (*p* < 0.01) [80]. Patients with septal flattening on CT have been shown to be associated with all-cause mortality (*p* = 0.002), PE-related mortality (*p* = 0.0067), and adverse clinical outcomes (*p* = 0.0008) [81]. Clinically, if a radiologist reports a D-shaped LV on the PE CT, the treating team should be alerted that this is not a trivial PE. Additional monitoring, ICU-level care, or consideration of thrombolysis is necessary if no contraindications are indicated. The specificity of the sign means that a false positive is unlikely; so if it is there, one should act on it.
- **Guide to Management:** Seeing the Movahed sign can guide management decisions. In chronic RV overload scenarios, it may push clinicians to more aggressively manage pulmonary pressures or consider interventions. For example, in a patient with an ASD and signs of RV overload, it supports the case for ASD closure if not done, before irreversible PH develops. In a patient with CTEPH, a persistent D-shaped LV on therapy might prompt referral for pulmonary endarterectomy or balloon pulmonary angioplasty. In post-operative cardiac surgery patients, if a transesophageal echo shows a D-shaped LV, anesthesiologists may adjust ventilation or support to reduce RV afterload (such as administering pulmonary vasodilators like inhaled nitric oxide).
- **Symptom Correlation:** The sign itself does not cause symptoms, but it reflects the pathophysiology leading to symptoms. A D-shaped LV means the LV is being compressed, which can reduce LV preload and cardiac output. This can contribute to hypotension or exercise intolerance. Patients with acute septal flattening (e.g., in massive PE) often have cardiogenic shock because the LV underfills as the septum bulges into it [82]. In chronic cases, the D shape contributes to reduced LV stroke volume during exertion (as the interventricular dependence limits LV filling on inspiration). Therefore, it is often present in patients with syncope or near-syncope in the setting of PH [6,7,83]. Recognizing it can emphasize the need to limit exertion and start therapy.
- **Monitoring Response to Therapy:** In serial imaging, resolution of or improvement in the D shape can be a positive sign. For example, after pulmonary endarterectomy for CTEPH, a follow-up CT or MRI might show that the septum is now more normal in curvature, indicating reduced RV pressure and improved hemodynamics. Conversely, the development of a D-shaped LV on follow-up of a PH patient signals progression. It can, thus, be a visual aid in monitoring.
- **Impact on Left Ventricular function:** When the septum bows leftward, it impairs LV filling and distensibility. This can cause a drop in LV stroke volume and blood pressure, especially in acute settings. In chronic settings, the LV may adapt to a smaller end-diastolic volume over time (hence, many PH patients have relatively small LV cavities on imaging). The clinical implication is that therapies to unload the RV (diuretics, vasodilators) can paradoxically improve systemic output by allowing the LV to re-expand. It also means that in PH patients, part of their heart failure symptoms (fatigue, low output) is due to this interventricular interaction. Some advanced therapies (septostomy in PH) even intentionally create an ASD to allow the septum to shift and decompress the RV, trading off a right-to-left shunt for improved left filling, thus underscoring how critical the septal position is [84,85,86].
- **Arrhythmias and Conduction:** Chronic distortion of the septum can be a substrate for arrhythmias. While not directly an implication of the D shape, PH patients with septal flattening often have RV hypertrophy and dilation, which can lead to arrhythmias like atrial flutter or fibrillation [79,87,88,89]. The D-shaped septum itself on imaging might hint at underlying changes in myocardial fiber orientation and strain that could predispose to arrhythmias.

In summary, a clinician who notes the Movahed sign on a patient’s imaging should interpret it as a red flag. In nearly all contexts, it implies that the patient’s condition is at a stage where the RV is no longer compensating well. Prognostically, it often correlates with higher morbidity and mortality, thus warranting closer follow-up and aggressive management. Therefore, its recognition is clinically valuable not just for diagnosis but also for risk stratification and guiding therapy.

## 9. Limitations, Controversies, and Debates

As a narrative review, this manuscript is subject to potential selection and reporting bias. The absence of systematic inclusion/exclusion criteria limits reproducibility.

Despite its clear significance, there are some limitations and points of debate regarding the Movahed sign:

**1. Sensitivity vs. Specificity:** A consistent theme is that the sign is highly specific but not very sensitive for RV overload [1,24]. Many patients can have significant RV strain without frank septal flattening. For example, mild to moderate PH may not produce a D-shaped LV, yet the patient still has RV dysfunction. Thus, relying on the Movahed sign alone to screen for RV overload would miss many cases. In Movahed’s own series, echocardiography detected other signs of RV overload (RV dilation, high PA pressure) even when the septal flattening was not obvious [2]. In CT studies of PE, the RV/LV diameter ratio can be abnormal while the septum still appears curved normally, reflecting the fact that RV dilation is often an earlier and more sensitive marker of RV overload than septal flattening [25]. Therefore, some have debated the utility of the sign as an early indicator since it appears late in the pathophysiologic cascade. However, others argue its high specificity makes it valuable as a confirmatory sign. This balance between sensitivity and specificity is well recognized: you should not rule out RV strain if the sign is absent, but if it is present, you can confidently rule in RV strain.

**2. Modalities Comparison—is one better at visualizing the sign?** There has been discussion about whether nuclear SPECT, echocardiography, or cross-sectional imaging is superior for detecting the D-shaped LV. Movahed’s initial finding was that SPECT caught the D shape more often than echo in his small sample. Echocardiography is highly observer-dependent and can sometimes miss flattening if the imaging plane is off-axis or if the acoustic window is poor. SPECT, on the other hand, provides a true short-axis slice every time (albeit at lower resolution) and might identify the sign in cases echo does not. CT and MRI, with their high resolution, should, in theory, be the most sensitive, but CT is usually only performed in select scenarios (like PE), and MRI, while the gold standard for structure, is not carried out routinely for all PH patients early on. A small controversy, or rather a practical point, is as follows: should we systematically check for this sign on all gated myocardial perfusion scans? Movahed and colleagues advocate yes, as it can unmask occult RV pressure overload in patients who came for evaluation of ischemia. Not all nuclear cardiologists were initially aware of this; over the past decade, awareness has increased.

**3. Flattening and Bowing—Quantification Challenges and Definitions:** Different literature sources use different criteria to define an abnormal septal contour. In echocardiography, an eccentricity index >1 at end systole is often used [3]. Some studies and expert reviews have proposed higher cutoffs, such as an EI >1.08 or >1.2, to increase specificity for RV pressure overload, especially in distinguishing patients with PAH from healthy controls. However, the most broadly accepted and guideline-endorsed threshold remains an EI > 1.0 [79]. In CT studies, some define any visible flattening as positive, while others use a qualitative grading. This lack of a uniform quantitative threshold can lead to interobserver variability. One study on CT reproducibility found only moderate agreement on identifying septal bowing, partly because of a lack of standardized images and criteria [77,90]. Technical nuances to measurements mean that some borderline cases might be labeled differently by different observers. Nonetheless, in obvious cases, the D shape is clear to all.

**4. Research and Controversies:** Research is ongoing on how best to integrate septal shape analysis into routine practice and risk algorithms. It is reasonable to argue that since it is so specific, it should be included in risk scores for PE or PH. However, its low sensitivity limits its independent utility. In an era of quantitation and AI, there could be interest in the automated detection of septal flattening on imaging. AI algorithms for echo or CT could flag a D-shaped ventricle automatically, potentially standardizing detection. As of now, human observation remains the mainstay.

Lastly, a limitation in the literature is that most information on this sign’s implications comes from echo and nuclear studies, with relatively fewer MRI-specific studies (though MRI findings are extrapolated from the same physiology). High-quality MRI and CT studies focusing on septal shape as a variable are now emerging, which will strengthen the evidence base [71,91,92].

In conclusion, the limitations of the Movahed sign are mostly about it being a later-stage marker (so not a catch-all screening tool) and minor issues of measurement and terminology. There is little controversy about its fundamental meaning; virtually all experts agree that a D-shaped left ventricle indicates significant RV overload. The debates lie in how early to look for it, how to quantify it, and ensuring it is not overlooked during imaging interpretation. As with many signs, increased awareness and standardized assessment can mitigate these issues. Overall, recognizing the sign and understanding its context are far more important than the minor debates around it.

## 10. Conclusions

The Movahed sign, a D-shaped left ventricle due to interventricular septal flattening, is a specific marker of RV overload seen across cardiac imaging modalities. Its recognition on MRI or CT should prompt evaluation for PH, PE, or other causes of RV strain. Though not a sensitive finding, its presence correlates with disease severity and adverse prognosis. Clinicians and imagers should routinely assess septal morphology as part of right heart evaluation.

## Figures and Tables

**Table 1:** Clinical cases of septal flattening observed on cardiac MRI or CT across various disease states.

Age/Gender	Imaging Modality	Key Findings	Clinical Diagnosis
61/Male	Cardiac MRI	Flattened or left-bowing septum during diastole; eccentricity index abnormal	Severe idiopathic pulmonary hypertension [23]
38/Female	Cardiac MRI	Enlarged RV, leftward bowing of ventricular septum	Atrial septal defect [23]
15/Female	Cardiac MRI	Crescentic LV with leftward septal bowing in systole; D-shape during systemic stress	Dextrose-transposition of the great arteries post atrial switch (chronic RV pressure overload) [23]
24/Female	Cardiac MRI	Leftward septal bowing of the right ventricle, which was accentuated during systole	Tricuspid regurgitation [23]
42/Female	Cardiac MRI	Flattening/leftwards septal bowing during diastole	Pulmonary regurgitation [23]
18/Male	Cardiac MRI	Hypertrophied right ventricle and septal leftwards bowing during early diastole	severe idiopathic pulmonary hypertension [23]
60/Male	Cardiac MRI	Inspiratory septal bowing during early diastole	Inflammatory pericarditis [23]
48/Male	Cardiac MRI	Leftwards septal shift during early systole and normal configuration of the interventricular septum at end diastole	Myocardial infarction and left branch block bundle [23]
21/Male	Cardiac MRI	Early systolic leftward septal motion and the sustained leftward motion during mid-late systole	Complete repair of tetralogy of Fallot [76]
35/Female	Cardiac MRI	Systolic anterior motion of the ventricular septum parallel to the posterior wall of the left ventricle with enlargement of the right ventricle	Sinus venous type ASD with moderate volume overload [76]
57/Male	Cardiac MRI	Diastolic flattening of the septal wall	Pulmonary thromboembolism [76]
63/Male	Cardiac MRI	Early diastolic septal flattening	Mitral stenosis [76]
54/Female	Cardiac MRI	Early diastolic septal flattening	Constrictive pericarditis [76]
63/Male	Cardiac MRI	Septum in early systole adopts a more inner position compared to that in end systole	Left bundle branch block [76]
57/Female	Cardiac CT	Four-chamber view shows septal bowing convex toward left ventricle	Pulmonary thromboembolism [77]
77/Female	Cardiac CT	Four-chamber view shows septal flattening	Pulmonary thromboembolism [75]
69/Male	Cardiac CT	Four-chamber view shows septal bowing convex toward the left ventricle	Pulmonary thromboembolism [75]
